# Interleukin-6 Levels in Patients With Diabetic Polyneuropathy

**DOI:** 10.7759/cureus.21952

**Published:** 2022-02-06

**Authors:** Debarati Chanda, Saswati Ray, Debjani Chakraborti, Sangita Sen, Asis Mitra

**Affiliations:** 1 Physiology, Jagannath Gupta Institute of Medical Science and Hospital, Kolkata, IND; 2 Physiology, Institute of Post Graduate Medical Education & Research, Kolkata, IND; 3 Internal Medicine, Ruby General Hospital, Kolkata, IND

**Keywords:** il-6, painful neuropathy, pathogenesis of dpn, diabetic polyneuropathy(dpn), type 2 diabetes

## Abstract

Introduction

Diabetic polyneuropathy (DPN) is a common chronic complication of type 2 diabetes. The pathogenesis of DPN is still debated, but proinflammatory cytokine mediators like interleukin-6 (IL-6) are possibly involved. We conducted this cross-sectional observational study to assess whether IL-6 levels increase in patients with DPN.

Materials and methods

This study was conducted at the Institute of Post Graduate Medical Education and Research Hospital in Kolkata, India, from 2016 to 2017. The study included 57 patients aged 30 to 60 years diagnosed with type 2 diabetes with neuropathy on clinical examination and nerve conduction study. Patients with neuropathy due to other causes were excluded. The study participants were assigned into one of four groups. Group 1 (n=15) served as healthy control patients, Group 2 (n=12) contained patients with type 2 diabetes without neuropathy, Group 3 (n=20) contained patients with type 2 diabetes with painful neuropathy, and Group 4 (n=10) contained patients with type 2 diabetes with painless neuropathy. We compared IL-6 levels between each group.

Results

There was no significant difference in serum IL-6 levels between healthy controls (Group 1) and patients with type 2 diabetes without neuropathy (Group 2). However, we noted a significant increase in serum IL-6 levels among patients with painful DPN (Group 3) compared to control groups. Interestingly, serum IL-6 levels were higher in patients with painful DPN (Group 3) than patients with painless DPN (Group 4).

Conclusions

IL-6 increases significantly in painful diabetic neuropathy patients compared to patients with diabetes with painless neuropathy and thus may have a role in the pathogenesis of pain in DPN. Serum IL6 level can be a potential noninvasive marker of painful DPN, and it can help distinguish painful DPN from other causes of pain in patients with diabetes.

## Introduction

Diabetes is a growing health problem globally, and type 2 diabetes (T2D) is the most common form of diabetes [1]. A common microvascular chronic complication of T2D is diabetic polyneuropathy (DPN) [2]. The pathogenesis of DPN is complex and multifactorial and includes an increase in levels of several cytokines such as tumor necrosis factor α (TNF-α) and interleukin-6 (IL-6) [3]. Interleukin 6 is a pleiotropic cytokine and a proinflammatory marker [4,5]. It is essential in the homeostasis of the peripheral nerve [6]. Elevated levels of IL-6 induce neuroinflammation leading to the development of pain in DPN [7]. Specific inflammatory mediators are significantly higher in painful DPN than in painless DPN. Inflammatory markers are also higher in T2D patients with peripheral neuropathy than those without peripheral neuropathy. According to Baka et al., a proinflammatory state might be the common denominator of pain and peripheral neuropathy in diabetes patients, but the inflammatory profiles seem to differ [8].

The role of IL-6 in DPN development has been evaluated and studied in several ways. However, data are limited on the role of serum IL-6 in DPN patients in India. Therefore, this study aimed to evaluate the relationship between IL-6 levels and the severity of DPN in patients with T2D.

## Materials and methods

We conducted this observational cross-sectional study at the Department of Physiology in association with the Department of Endocrinology at the Institute of Post Graduate Medical Education and Research Hospital in Kolkata, India, from February 2016 to January 2017. The study included patients aged 30 to 60 years diagnosed with T2D according to the American Diabetes Association criteria [9]. Patients were included if they had evidence of neuropathy from patient history and clinical examinations, including vibration perception threshold nerve conduction studies. We excluded patients with neuropathy due to other causes, including chemotherapy, nutritional deficiency, hypothyroidism, and chronic kidney disease. We also excluded patients treated with isoniazid or hydralazine. Patients with autoimmune diseases, malignancy, or pregnancy were also excluded.

Fifty-seven patients were included. All participants provided written informed consent. We sorted the participants into four study groups. Group 1 contained 15 healthy control patients, Group 2 contained 12 patients with T2D but no neuropathy, Group 3 contained 20 patients with T2D and painful neuropathy and Group 4 contained 10 patients with T2D and painless neuropathy. All groups were matched for age and sex.

We took detailed histories and recorded patient age, body mass index (BMI), duration of T2D, and glycated hemoglobin (HbA1c) levels and assessed patients according to the Toronto Clinical Scoring system (TCS) to determine the severity of their neuropathy [10]. Of a total score of 19, the TCS grades are defined as follows: scores of 0 to 5 indicate no neuropathy, 6 to 8 indicate mild neuropathy, 9 to 11 is moderate neuropathy, and scores ≥ 12 correlate to severe neuropathy. The TCS includes sensory and motor symptoms like pin sensitivity, vibration sense, deep tendon reflexes, muscle power, vibration perception threshold, and a motor nerve conduction test.

To measure IL-6 levels, we drew 5 mL of blood from each participant. We separated the plasma via centrifugation then used the Human IL-6 enzyme-linked immunoassay (ELISA) kit to determine IL-6 levels (RayBiotech Life, Inc., Peachtree Corners, GA, USA) [11].

The hospital's Institutional Ethics Committee approved the design of the study (Approval No.: Inst/IEC/1307). No patient identifying information was collected or revealed in the data.

Statistical analysis

We calculated mean ± SD for each measurement. We used one-way analysis of variance and the Student’s t-test and correlation analysis using GraphPad PRISM (GraphPad Software, San Diego, CA, USA). We considered p < 0.05 as statistically significant.

## Results

**Figure 1:**
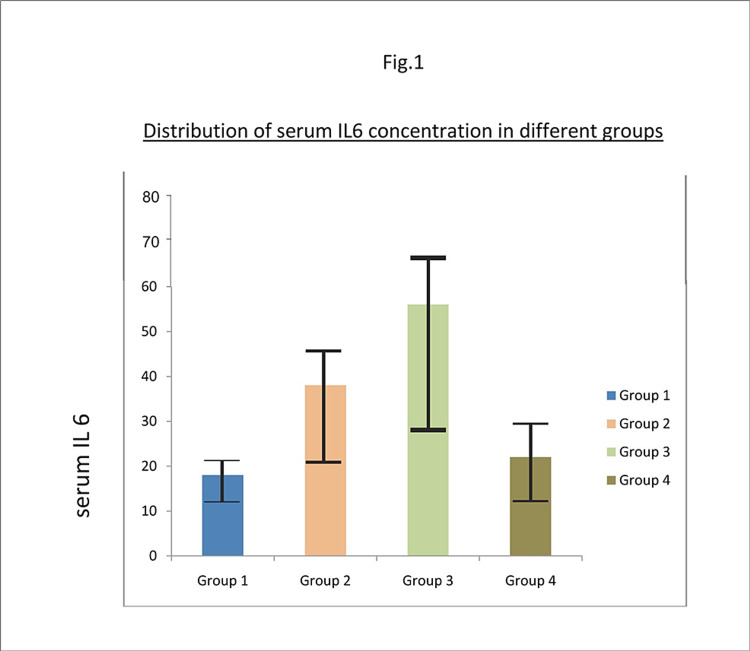
Distribution of Serum IL-6 concentration in different groups IL-6: Interleukin-6

Table 1 presents all study results. The mean ages of patients in Groups 1, 2, and 3 were similar, but the mean age of patients in Group 4 was higher than the other groups. Mean BMI is presented in Table 1, and the mean BMI of Group 4 was significantly higher than the other groups. The HbA1c levels (Table 1)slightly increased from Group 1 through Group 4, with Group 4 retaining the highest HbA1c values. The TCS of Group 3 was 13.1 ± 1.41, and TCS for Group 4 was 22.6 ± 2.836. Participant plasma IL-6 levels were statistically significantly higher in Group 3 than Group 1 (p<0.01), Group 2 (p<0.01), and Group 4 (p<0.01; Figure 1). Plasma IL-6 levels were higher in patients with T2D and painful DPN than those with painless DPN. Plasma IL-6 levels did not correlate with DPN severity.

**Table 1:** Patient characteristics, neuropathy, and IL-6 levels a: Significantly higher than Group 1 (p<.001); b: Significantly higher than Group 2 (p<0.01),  c: Significantly higher than Group 4 (p<0.01) BMI: Body mass index; HbA1c: Glycated hemoglobin; IL-6: Interleukin-6; SD: Standard deviation; TCS: Toronto Clinical Scoring System

Parameters	Group 1	Group 2	Group 3	Group 4
Age in years (mean ± SD)	45.9 ± 9.0	45.1 ± 9.6	47.3 ± 8.7	54.7 ± 4.1
Duration in years (mean ± SD)	0	1.417 ± 0.41	7.55 ± 2.87	17.66 ± 2.71
BMI kg/m^2 ^(mean ± SD)	22.57 ± 1.806	25.27 ± 1.115	22.41 ± 3.364	27.11 ± 1.649
HbA1c % (mean ± SD)	5.407 ± 0.337	7.150 ± 0.504	8.24 ± 2.013	9.33 ± 1.857
TCS (mean ± SD)	0	0	13.1 ± 1.41	22.6 ± 2.836
IL-6 pg/ml (mean ±SD)	17.43 ± 7.674	32.91 ± 13.44	52.34 ± 26.28^a,b c^	22.75 ± 11.9

## Discussion

We conducted this study to explore the relationship between serum IL-6 concentration and DPN in T2D patients in India. The role of IL-6 in DPN development has been previously reported in the literature. Zheng et al. showed serum TNF-α, and IL-6 increased significantly in patients with T2D compared to control patients [12]. This finding is similar to our observation. Herder et al. found that TNF-α and IL-6 were predictors of incident distal sensorimotor polyneuropathy (DSPN), whereas interleukin-1 receptor antagonist and intercellular adhesion molecule-1 were related to the progression of DSPN [10]. Our results align with these findings.

Magrinelli et al. reported that serum IL-6 was elevated in 44% of their patients and significantly inversely correlated with sensory nerve action potentials and compound muscle action potentials. They suggest IL-6 has a role in peripheral nerve axonal damage [13]. We observed IL-6 levels were not significantly different between healthy control patients and T2D patients. However, IL-6 in T2D patients with DPN was significantly higher than in patients with T2D without neuropathy.

Zin et al. showed that both serum TNF-α and IL-6 were significantly higher in patients with T2D than healthy control patients [2]. Our study noted a nonsignificant increase in mean IL-6 levels in T2D patients compared to healthy controls [2]. Herder et al. conducted an additional large population-based study that explored the correlation between inflammatory markers and painful DSPN in older individuals [14]. The study population consisted of men and women aged 61 to 82 years with painless (n = 337) and painful DSPN (n = 54). After adjustment for age and sex, they found positive associations between serum concentrations of IL-6 and painful DSPN (p = 0.004). We found similar results where IL-6 levels were positively correlated with painful DPN rather than neuropathy severity according to TCS.

Our study had several important limitations. This was a single-center study with a relatively small population size. The circulating IL-6 levels might be influenced by other factors like subclinical chronic inflammatory conditions. Exploring the IL-6 level or its expression directly in nervous tissues by nerve biopsy might offer data that are better correlated with the clinical and neurophysiological findings, but these approaches would have been more invasive. Another limitation was the study’s cross-sectional design, so we could not separate risk or causative factors from a coincidental rise in serum IL-6 levels in different study population groups. Finally, we did not evaluate autonomic neuropathy - another feature of peripheral nerve damage in patients with T2D. Future extensive multicentered prospective studies are required to assess the role of IL-6 in the pathogenesis of DPN and whether modulation of IL-6 may have a therapeutic role in DPN.

## Conclusions

According to our results, the pro-inflammatory marker IL-6 is highly correlated with the severity of pain rather than the severity of neuropathy score. Interleukin-6 tended to be higher in T2D patients than healthy controls and even higher in T2D patients with painful neuropathy but lower in patients with painless DPN. Interleukin-6 plays a crucial role in the development and progression of neuropathy, and the increase in the concentration of IL-6 with the severity of painful neuropathy suggests that IL-6 may mediate the development of painful DPN. Interleukin-6 is a noninvasive marker of painful DPN, and it can be used as a supportive diagnostic test to rule out other causes of limb pain in patients with T2D.
